# Community-based epidemiological study on the prevalence and health status of Spondyloarthritis in residents of Wuyuan County, China, 2023

**DOI:** 10.1515/rir-2026-0001

**Published:** 2026-03-30

**Authors:** Shichao Yu, Xiuling Zhang, Zhijie Liu, Dunwu Wu, Yuanjia Pan, Jinying Wang, Jinghong Li, Libo Zha, Yumin Dong, Senmiao Zhang, Guoliang Wu, Zhongpei Wang, Guixing Dong, Junbo Zhu, Nan Wang, Xinwang Duan, Zhirao Huang

**Affiliations:** Department of Rheumatology and Immunology, Wuyuan County Maternal and Child Health Hospital, Shangrao 333200, Jiangxi Province, China; Department of Rheumatology and Immunology, The Second Affiliated Hospital of Nanchang University, Nanchang 330006, Jiangxi Province, China; Tuochuan Township Health Center, Wuyuan County, Shangrao 333200, Jiangxi Province, China; Taibai Town Health Center, Wuyuan County, Shangrao 333200, Jiangxi Province, China; Duanxin Township Health Center, Wuyuan County, Shangrao 333200, Jiangxi Province, China; Zhentou Town Health Center, Wuyuan County, Shangrao 333200, Jiangxi Province, China; Qiukou Central Health Center, Wuyuan County, Shangrao 333200, Jiangxi Province, China

**Keywords:** spondyloarthritis, prevalence, health status, epidemiological investigation

## Abstract

**Background and Objective:**

The prevalence of Spondyloarthritis (SpA) in China is approximately 1%, with ankylosing spondylitis (AS) at 0.25%. However, community-based incidence data remain scarce and are subject to medical reporting bias. This study aimed to investigate the prevalence of SpA and the health status of patients in Wuyuan County, to provide evidence for the national “Rural Health Project for Ankylosing Spondylitis”.

**Methods:**

A cross-sectional survey was conducted from August to December 2023 using the WHO COPCORD methodology. A multi-stage stratified random cluster sampling method was employed, including 16, 996 residents. A screening questionnaire based on assessment of Spondyloarthritis international society (ASAS) criteria was used, and SpA diagnoses were confirmed by rheumatologists. Data analysis included Poisson distribution sample size estimation, Mann-Whitney *U* test, and Bootstrap proportion estimation (SPSS v29.0 and R v4.3.2).

**Results:**

The estimated prevalence of SpA among residents of Wuyuan County in 2023 was 0.14%. A total of 24 patients were diagnosed with SpA (16 males and 8 females). Half of these cases were previously undiagnosed and demonstrated a significant diagnostic delay (*P* = 0.021). Male patients exhibited more severe functional impairment, as indicated by higher bath ankylosing spondylitis functional index (BASFI) scores compared to their female counterparts (3.29 *vs*. 1.44, *P* < 0.05).

**Conclusion:**

Community screening revealed a significant burden of SpA in rural China, underscoring the need to strengthen primary healthcare resource allocation.

## Introduction

Spondyloarthritis (SpA) refers to a group of chronic, progressive, and long-lasting autoimmune diseases. These disorders often lead to inflammation, deformity, and ankylosis of the spine and peripheral joints.^[1]^ In China, the prevalence of SpA is approximately 1%, with ankylosing spondylitis (AS) accounting for about 0.25%.^[2]^ There is limited reported data on the incidence of SpA in large-scale Chinese populations, and most existing epidemiological data are derived from medical institutions, which may carry a risk of healthcare-seeking bias.^[3]^ In 2017, we conducted the “Epidemiological Survey of Rheumatic and Immunological Diseases Among Urban and Rural Residents of Wuyuan County”, which established a baseline SpA prevalence of 0.03%.^[4]^ To understand the epidemiological trends of SpA in Wuyuan County over the past seven years, we conducted another cross-sectional survey based on the WHO-recommended COPCORD (Community-Oriented Program for Control of Rheumatic Diseases) methodology. This study aims to further investigate the current status and characteristics of SpA among community residents in Wuyuan County, determine the changes in patients’ health status, provide a theoretical basis for targeted SpA prevention and control strategies, and support the implementation of the national “Ankylosing Spondylitis Healthy Rural Project”.^[5]^

## Methods

### Study Population and Design

**Figure 1:**
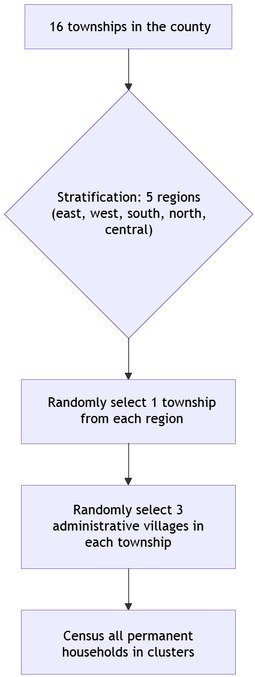
Flowchart of multistage stratified random cluster sampling for SpA survey. SpA, Spondyloarthritis.

A community-based cross-sectional survey was conducted in Wuyuan County, Jiangxi Province, from August 11 to December 1, 2023. The target population consisted of permanent residents (living in the area for ≥ 6 months), with a base population of 334,020. Using the Poisson distribution (*P*_0_ = 0.004, *P* = 0.003), a minimum sample size of 13,330 was estimated. Accounting for a 20% loss to follow-up, the sample was expanded to 15,996, and ultimately 16,000 individuals were enrolled. The multi-stage stratified random cluster sampling framework from the 2017 study^[4]^ was adopted, covering five townships: Taibai Town, Duanxin Township, Zhentou Town, Qiukou Town, and Tuochuan Township (Figure 1).

### Screening and Diagnostic Procedures

Data Collection: Household surveys were conducted *via* face-to-face interviews, WeChat-based questionnaires, or telephone interviews. Village doctors who had passed standardized training and assessment administered the initial screening. Assistance was provided to those unable to self-complete the questionnaire.

### Screening Tools

Initial Screening: A questionnaire based on the 1984 New York Criteria for AS and the 2009 ASAS criteria for inflammatory back pain was used to assess eight symptoms (*e.g*., nocturnal pain, morning stiffness).^[6]^ Participants reporting ≥ 4 positive symptoms proceeded to secondary screening (Table 1). Individuals with a prior diagnosis of AS were directly referred to the next stage.

**Table 1:** Primary screening questionnaire for SpA risk

Screening Item	Yes	No
Existing SpA diagnosis (if “No,” continue below)		
1. Onset age < 40 years		
2. Insidious onset: Gradual back pain, not acute		
3. Nocturnal pain: Pain that awakens the patient at night		
4. Worsening pain at rest with stiffness		
5. Improvement with activity		
6. Duration ≥ 3 months		
7. Limited lumbar flexion		
8. History of arthritis, enthesitis, uveitis, dactylitis, psoriasis, or inflammatory bowel disease.		

Result: Suspected SpA if ≥ 4 items positive. SpA, Spondyloarthritis.

​Secondary Screening: Additional imaging and laboratory parameters were evaluated, including radiographic sacroiliitis and elevated High-sensitivity C-reactive protein (hs-CRP)/erythrocyte sedimentation rate (ESR). Those with ≥ 4 symptoms and positive imaging or laboratory findings were classified as highly suspected cases (Table 2).

**Table 2:** Secondary screening questionnaire for SpA risk

Screening Item	Yes	No
Items 1–8 from Table 1		
9. X-ray evidence (sacroiliitis grade II–IV) and/or elevated ESR/ hs-CRP		

Result: High suspicion if ≥ 4 items from 1–8 plus item 9 positive. SpA, Spondyloarthritis; ESR, erythrocyte sedimentation rate; hs-CRP, High-sensitivity C-reactive protein.

Diagnostic Confirmation: Rheumatologists from tertiary hospitals applied the 2009/2011 ASAS classification criteria and the 1984 New York Criteria for definitive diagnosis (Table 3).^[7]^

**Table 3:** Three-stage screening protocol ​

Stage	Tool	Positive Criteria	Executor
Primary	Initial questionnaire	≥ 4 positive items	Village physicians
Secondary	Secondary questionnaire	Initial screen positive + objective evidence	County rheumatologists
Confirmation	2009/2011 ASAS or 1984 NY criteria	Diagnostic fulfillment	Tertiary specialists

### Training and Ethics

Survey personnel received standardized training covering disease recognition, common symptoms and signs of SpA, interpretation of findings, survey procedures, statistical methods, data entry and management, and quality control (pass rate: 98%). The study was approved by the Ethics Committee of Wuyuan County Maternal and Child Health Hospital (Approval No. 2023NO. 03). Informed consent was integrated into the questionnaire, and participation was based on the voluntary principle.

### Statistical Analysis

Data were double-entered using Epi Data 3.0 and analyzed with SPSS 29.0 and R 4.3.2. Continuous variables with nonnormal distribution are presented as median (interquartile range, IQR) deviation and compared using the Mann-Whitney *U* test. Categorical variables are reported as frequency (proportion, %) and analyzed by the *χ*^2^ test. Bootstrap resampling (1000 repetitions) was applied to estimate the 95% confidence intervals (95% CI) for proportions. A two-sided *P*-value < 0.05 was considered statistically significant.

## Result

### Participant Characteristics

The method of acquiring the study participants in this project was consistent with the 2017 survey: a stratified sampling method was adopted, selecting one township from each of the eastern, western, southern, northern, and central regions of Wuyuan County, namely Taibai Town (27.9%), Duanxin Township (20.2%), Zhentou Town (19.8%), Qiukou Town (16.6%), and Tuochuan Township (15.2%). An additional 31 individuals were categorized as “other” due to unclear address information. A total of 18, 851 questionnaires were collected during the initial screening. The permanent resident population in the selected administrative villages at the end of 2023 was 21,089, resulting in a questionnaire response rate of 89.39%. After excluding questionnaires from participants who did not provide informed consent and those with duplicate submissions, 16,982 valid questionnaires were retained, yielding a validity rate of 90.09%. The valid sample was predominantly male, with the majority concentrated in the 19–64 age group. Detailed demographic characteristics are presented in Table 4.

**Table 4:** Demographics of primary screening participants (n = 16,982) ​

Variable	Category	*n*	%
Gender	Male	9077	53.5
	Female	7905	46.5
Township	Taibai	4739	27.9
	Duanxin	3433	20.2
	Tuochuan	2585	15.2
	Qiukou	2827	16.6
	Zhengtou	3367	19.8
	Other	31	0.2
Age group	Adolescents < 18 years	2769	16.3
	Adults 19–64 years	11,285	66.5
	Older adults ≥ 65 years	2928	17.2

A secondary screening was conducted among individuals who had either been previously diagnosed with SpA or had met the primary screening criteria (≥ 4 items), resulting in the collection of 442 questionnaires. Among these, females constituted the majority (58.4%). Geographically, Tuochuan Township accounted for the highest proportion (44.3%). In terms of age distribution, the adult group (51.8%) and the elderly group (47.1%) were predominant. Details are presented in Table 5.

**Table 5:** Demographics of secondary screening participants (n = 442) ​

Variable	Category	*n*	%
Gender	Male	184	41.6
	Female	258	58.4
Township	Taibai	73	16.5
	Duanxin	74	16.7
	Tuochuan	196	44.3
	Qiukou	47	10.6
	Zhengtou	52	11.8
Age group	Adolescents < 18 years	5	1.1
	Adults 19–64 years	229	51.8
	Older adults ≥ 65 years	208	47.1

### SpA Suspicion Rates

Tables 6 and 7 present the distribution and intergroup comparisons of suspected SpA cases by different characteristics in the primary and secondary screenings, respectively. Among the 16,982 participants included in the primary screening, 459 were identified as suspected SpA cases, yielding an overall suspected proportion of 2.7% (459/16,982), with a 95.0% confidence interval (CI) of 2.5%–3.0%. The suspected proportion was 2.4% (214/9077) in males and 3.1% (245/7905) in females, showing a statistically significant difference between genders (*P* < 0.01). Geographically, Tuochuan Township had the highest proportion of suspected cases at 7.8% (202/2585). Data for the remaining townships are shown in Table 6, and the differences among townships were also statistically significant (*P* < 0.001). In terms of age distribution, the elderly group (≥ 65 years) had the highest proportion of suspected cases at 6.9% (202/2928). Data for the other age groups are provided in Table 6, and the differences among age groups were statistically significant (*P* < 0.001).

**Table 6:** SpA suspicion rates in primary screening by demographics (n = 16,982)

Variable	Number of individuals investigated	Number of suspected cases	% (95% CI)	χ^2^	*P*
Gender				8.839	0.003
Male	9077	214	2.4 (2.1–2.7)		
Female	7905	245	3.1 (2.8–3.5)		
Township				309.370	< 0.001
Taibai	4739	76	1.6 (1.2–2.0)		
Duanxin	3433	60	1.7 (1.3–2.2)		
Tuochuan	2585	202	7.8 (6.8–9.0)		
Qiukou	2827	65	2.3 (1.8–2.9)		
Zhengtou	3367	54	1.6 (1.1–2.0)		
Other	31	2	6.5 (0.0–17.2)		
Age group				270.947	< 0.001
Adolescents < 18 years	2769	6	0.2 (0.0–0.4)		
Adults 19–64 years	11,285	251	2.2 (1.9–2.5)		
Older adults ≥ 65 years	2928	202	6.9 (6.0–7.9)		

SpA, Spondyloarthritis.

**Table 7:** SpA suspicion rates in secondary screening by demographics (n = 442) ​

Variable	Number of individuals investigated	Number of suspected cases	% (95% CI)	χ^2^	*P*
Gender				2.757	0.097
Male	184	23	12.5 (8.0–17.5)		
Female	258	20	7.8 (4.6–11.2)		
Township				27.307	< 0.001
Taibai	73	14	19.2 (10.1–28.6)		
Duanxin	74	15	20.3 (10.8–29.8)		
Tuochuan	196	6	3.1 (1.0–5.5)		
Qiukou	47	3	6.4 (0.0–14.3)		
Zhengtou	52	5	9.6 (2.2–17.6)		
Age group				4.357	0.113
Adolescents < 18 years	5	1	20.0		
Adults 19–64 years	229	28	12.2 (8.1–16.5)		
Older adults ≥65 years	208	14	6.7 (3.4–10.3)		

SpA, Spondyloarthritis.

Among the 459 suspected SpA cases identified in the primary screening, 442 participated in the secondary screening. Of these, 43 met the criteria for further analysis by either being previously diagnosed or by meeting the primary screening criteria (≥ 4 items) in addition to having one or more supporting indicators (Table 6, 7).

### Basic Characteristics of Diagnosed Patients

A total of 24 patients were diagnosed with SpA in this study, including 22 cases of AS, one case of juvenile spondyloarthritis (JSpA), and one case of psoriatic arthritis (PsA).

Among the diagnosed patients, 16 were male and 8 were female. We compared basic characteristics between the 12 previously diagnosed patients (50%) and the 12 newly identified patients (50%) who had not previously sought medical attention. The time from symptom onset to first medical consultation was significantly longer in the undiagnosed group (15.58 ± 9.811 years) compared to the previously diagnosed group (6.58 ± 4.400 years) (*P* < 0.05). The overall and comparative characteristics of the 24 confirmed patients are detailed in Tables 8 and 9.

**Table 8:** Summarizes the basic characteristics of confirmed patients (n = 24)

Hallmark	Median/n	IQR/%
Cigarette smoking, *n* (%)	11	45.83%
Drinking wine, *n* (%)	9	37.50%
HLA-B27 positive, *n* (%)	18	75.00%
Previously treated or not, *n* (%)	17	70.83%
Age, median (IQR)	48.50	(35.75–57.00)
BMI, median (IQR)	23.07	(21.16–24.61)
Laboratory tests		
RBC (×1012/L), median (IQR)	4.39	(4.14–4.83)
Hb (g/L), median (IQR)	134.50	(124.25–152.50)
Lymphocytes (×10^9^/L), median (IQR)	1.56	(1.23–2.06)
CRP (mg/L), median (IQR)	1.79	(1.07–11.15)
ESR (mm/1h), median (IQR)	15.50	(13.00–23.25)

HLA-B27, human leukocyte antigen B27; BMI, body mass index; CRP, C-reactive protein; ESR, erythrocyte sedimentation rate; RBC, red blood cell count; Hb, Hemoglobin; IQR, interquartile range.

**Table 9:** Comparison of characteristics between undiagnosed and previously diagnosed patients (n = 24)

Characteristic	No consultation Group (*n* = 12)	Previously Diagnosed Group (*n* = 12)	*P*
Female, *n* (%)^#^	5 (41.7)	3 (25.0)	0.385
Age ≥ 65 years, *n* (%)^#^	2 (16.7)	0 (0.0)	0.086
Family Income < ¥50,000, *n* (%)	9 (75.0)	5 (41.7)	0.098
HLA-B27 Positive, *n* (%)	8 (66.7)	11 (91.7)	0.121
Diagnostic Delay, years, Median (IQR)	13.00 (5.50–22.00)	8.00 (2.75–11.75)	0.021^*^

# denotes that the expected cell count was < 5, and the likelihood ratio test result was used; ^*^indicates *P* < 0.05. IQR, interquartile range.

### Health Status of Diagnosed Patients

A comprehensive health assessment was conducted on the 24 diagnosed patients using multiple instruments, including: SF-36, EQ-5D, BASDAI, BASFI, ASDAS-CRP, ASDAS-ESR. Among these, only the BSpAFI score was significantly higher in male patients compared to females. No other statistically significant differences were observed between genders in the remaining assessments. Detailed results are presented in Table 10.

**Table 10:** Comparison of health status and gender of confirmed patients (n = 24)

Evaluation projects	All patients (*n* = 24)	Male (*n* = 16)	Female (*n* = 8)
SF-36	53.55 (34.97–68.25)	54.92 (36.09–73.22)	46.78 (33.93–64.42)
EQ-5D	0.75 (0.66–0.89)	0.72 (0.61–0.86)	0.80 (0.73–0.93)
BASDAI	2.35 (1.40–3.73)	2.45 (1.40–3.95)	2.35 (1.50–2.90)
BASAFI	2.50 (1.03–3.55)	2.75 (1.50–4.95)^*^	1.10 (0.70–2.40)
ASDAS-CRP	1.64 (1.30–3.00)	1.61 (1.26–3.38)	1.64 (1.28–2.62)
ASDAS-ESR	2.24 (1.80–2.86)	2.18 (1.70–3.16)	2.30 (1.87–2.58)

* means *P* < 0.05.

## Discussion

This study, through a 2023 community-based cross-sectional survey, revealed the prevalence and health status of SpA among residents of Wuyuan County. The prevalence of SpA among Wuyuan County residents in 2023 was 0.14% (24 out of 16,982), which is higher than the baseline prevalence of 0.03% in 2017.^[4]^ This observed increase in prevalence is likely multifactorial. A key contributing factor is the optimization of screening methods in the current study, which integrated the modern ASAS criteria, potentially improving case detection compared to the 2017 survey. Other possible factors include increased health awareness among residents or a genuine rise in the disease burden.

Looking at the results from the screening phase, the suspected SpA rate showed variations across gender, age, and geographical distribution. The suspected rate was higher in females (3.1% *vs*. 2.4% in males), which differs from the traditional view that SpA predominantly affects males.^[8]^ This discrepancy is likely attributable to a combination of factors. First, it is well-documented that the clinical presentation of SpA differs by gender, with females more frequently exhibiting a higher burden of peripheral manifestations, such as arthritis and enthesitis^[9]^, which can contribute to underdiagnosis. Second, a degree of self-reporting bias, where in one gender may be more likely to report symptoms, cannot be excluded. The elderly group (≥ 65 years) had the highest suspected rate (6.9%), significantly higher than that of young and middle-aged adults, suggesting potential longterm diagnostic delays or symptom masking by comorbidities in rural areas.^[10,11]^ Geographically, Tuochuan Township had an exceptionally high initial suspected rate (7.8%), but it decreased significantly after re-screening. It is hypothesized that a combination of insufficient healthcare resources and local environmental factors, such as the damp and cold climate known to exacerbate rheumatic symptoms^[12]^, may contribute to difficulties in early identification in this area.

Among the 24 confirmed SpA patients, 50% were previously undiagnosed cases, with a diagnostic delay as long as 15.58 ± 9.81 years. A high proportion came from low-income households (75%), confirming that statistics based on healthcare institutions underestimate the true disease burden. Health status assessment showed that male patients had more severe functional impairment (higher BASFI scores). Overall, patients had low quality of life indicator scores (*e.g*., SF-36: 51.85 ± 19.03 points), which may be related to lower economic status and severe damage caused by diagnostic delays. This suggests that the health status of SpA patients in Wuyuan County needs improvement.

The findings of this study should be interpreted in the context of several limitations. First, given the inherent complexity of SpA diagnosis, some cases may have gone undetected despite the multi-stage screening protocol. Second, the generalizability of our findings is constrained by the study's single-region design and the limited number of confirmed cases. Furthermore, the cross-sectional nature of the study precludes the establishment of causal relationships. Therefore, larger, multicenter prospective studies are warranted to validate these preliminary findings.

In conclusion, this community survey indicates that the prevalence of SpA in Wuyuan County in 2023 is higher than in 2017, and half of the patients did not receive timely diagnosis and treatment. It is recommended to leverage the “Ankylosing Spondylitis Healthy Village Project” to enhance screening in remote areas, provide training for primary care doctors, and integrate resources. These measures aim to reduce diagnostic delays and improve patients’ quality of life.
